# Oleuropein reduces anxiety-like responses by activating of serotonergic and neuropeptide Y (NPY)-ergic systems in a rat model of post-traumatic stress disorder

**DOI:** 10.1080/19768354.2018.1426699

**Published:** 2018-03-13

**Authors:** Bombi Lee, Insop Shim, Hyejung Lee, Dae-Hyun Hahm

**Affiliations:** a Acupuncture and Meridian Science Research Center, College of Korean Medicine, Kyung Hee University, Seoul, Republic of Korea; b Department of Physiology, College of Medicine, Kyung Hee University, Seoul, Republic of Korea; c The Graduate School of Basic Science of Korean Medicine, College of Korean Medicine, Kyung Hee University, Seoul, Republic of Korea

**Keywords:** Oleuropein, post-traumatic stress disorder, single prolonged stress, anxiety, serotonin

## Abstract

Post-traumatic stress disorder (PTSD) is a stress-related mental disorder caused by traumatic experiences. This psychopathological response to traumatic stressors induces anxiety in rats. Oleuropein (OLE), a major compound in olive leaves, reportedly possesses several pharmacological properties, including anti-cancer, anti-diabetic, and anti-atherosclerotic and neuropsychiatric activities. However, the anxiolytic-like effects of OLE and its mechanism of action in PTSD are unclear. The present study used several behavioral tests to examine the effects of OLE on symptoms of anxiety in rats after a single prolonged stress (SPS) exposure by inhibiting the hypothalamic-pituitary-adrenal axis. Male Sprague Dawley rats received OLE (10, 50 and 70 mg/kg, i.p., once daily) for 14 days after SPS exposure. Daily OLE (70 mg/kg) administration significantly increased the number and duration of open arm visits in the elevated plus maze (EPM) test, reduced the anxiety index and grooming behavior in the EPM test, and increased the time spent and number of central zone crossings in the open field test. OLE also blocked the SPS-induced decrease in hippocampal serotonin and neuropeptide Y expression in hippocampus. These findings suggest that OLE has anxiolytic-like effects on behavioral and biochemical symptoms similar to those observed in patients with PTSD.

## Introduction

Post-traumatic stress disorder (PTSD), a chronic syndrome triggered by exposure to trauma, is characterized by symptoms of intrusive re-experiencing, physiological and psychological hyperarousal, affective numbing, and avoidance of trauma-related stimuli (Kozlovsky et al. 2009). The hallmark symptoms of PTSD includes nightmares, hyperarousal, chronic fear, and emotional numbing, leading to a profound social burden due to high rates of comorbidity with major depression, suicide, and psychosocial or occupational impairment (Ji et al. 2014). The symptoms of PTSD are believed to reflect trauma-induced changes that lead to long-term dysfunctional stress regulation and inappropriate adaptation in the hypothalamic-pituitary-adrenal (HPA) axis (George et al. 2013). Many studies have shown that inappropriate adaptation of the HPA axis can lead to pathological states of PTSD, producing serious changes in affective behavior that are indicative of or consistent with anxiety-like symptoms (Shea et al. 2005).

The leaves of the olive tree (Olea europeaea L.) have long been used in folk medicines in Mediterranean countries such as France, Greece, Italy, Morocco, Spain, Tunisia and Turkey (Hadrich et al. 2016). Olive leaves have been used in the human diet in the form of extracts, herbal teas, and powders, and they contain several potentially bioactive compounds that may having anti-oxidant, anti-hypertensive, anti-atherogenic, anti-inflammatory, hypoglycemic, and anxiolytic-like effects (Kaeidi et al. 2011). Oleuropein (OLE), a major phenolic compound in olive leaves, is responsible for these pharmacological properties (Hadrich et al. 2016). It improves multiple physiological actions, produces various pharmacological actions in the central nervous system, and has neuroprotective effects *in vitro* and *in vivo* (Dekanski et al. 2011; Pourkhodadad et al. 2016). OLE supplementation at high concentrations significantly decreased body weight, decreased the leptin concentration, and modulated the expression of genes related to obesity in male C57BL/6JOlaHsd mice; moreover, it stimulated the high cholesterol diet-induced inhibition of AMP-activated protein kinase (AMPK) in epididymal adipose tissue (Hadrich et al. 2016). It has also been shown that the administration of OLE before ischemia-reperfusion in the CA1 of hippocampus area reduced oxidative stress (Dekanski et al. 2011) and had neuroprotective effects against colchicine-induced cognitive dysfunction and oxidative damage in rats (Pourkhodadad et al. 2016). Thus, OLE, as an anti-stress supplement, may be valuable for the prevention of trauma- and stress-related disorders, such as PTSD. However, it is unknown whether treatment with OLE can improve anxiety-like symptoms following single prolonged stress (SPS) exposure in rats. The present study investigated the medicinal impacts of OLE on anxiety-related behaviors in rats exposed to SPS using the elevated plus maze (EPM) test and open field test (OFT), representing the core symptom of PTSD-related abnormalities. Moreover, we examined how the behavioral effects were associated with the serotonergic and neuropeptide Y (NPY)-ergic system in the brain as an underlying mechanism.

## Materials and methods

### Animals and OLE administration

The present study utilized adult male Sprague-Dawley (SD) rats weighing 200–220 g (6 week-old, Samtako Animal Company; Seoul, Korea). All rats were housed in a limited access rodent facility with five rats per polycarbonate cage. The room controls were set to maintain the temperature at 22 ± 2°C and the relative humidity at 55% ± 15%, the cages were lit by artificial light for 12 h each day, and sterilized drinking water and a standard chow diet were supplied ad libitum throughout the experiments. All efforts were made to minimize the number and suffering of animals.

OLE (20, 50 and 70 mg/kg, body weight, OLE; Sigma-Aldrich Chemical Co. St. Louise, MO, USA) and the positive drug fluoxetine (10 mg/kg, FLX, fluoxetine hydrochloride; Sigma) were administered by intraperitoneally (i.p.) in a volume of 1 ml/kg for 14 days after SPS procedure. The standard doses of OLE and FLX in the rat and considering the long-term treatment used in the present study was based on previous study (Hadrich et al. 2016). The OLE and FLX were dissolved in 0.9% physiological saline (SAL) before use.

### Single prolonged stress

**Figure 1.**
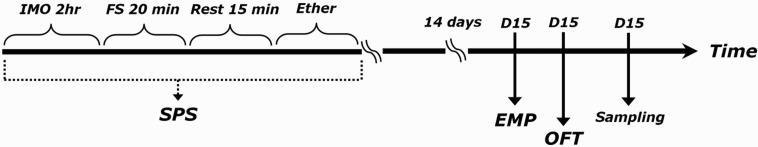
Experimental schedules for developing SPS-induced anxiety-like behaviors, and OLE treatment in the rats. Different groups of rats (*n* = 6 or 7 animals per group) were used for all experiments.

Briefly, rats were first immobilized for 2 h on restraint tubes (20 cm height, 7 cm diameter). Following immobilization, rats were immediately subjected to forced swim for 20 min in a plexiglass cylinder (50 cm height, 20 cm diameter) filled to two-thirds with 24°C fresh water. The animals were dried and allowed to recuperate for 15 min and then exposed to ether vapor until loss of consciousness. The following parameters were measured to monitor the effects of the development of psychiatric disorders by SPS model of PTSD: changes in body weight gains and serum corticosterone (CORT) levels. Testing for anxiety-like behavior was done 24 h after the end of the undisturbed condition. The entire experimental schedules of SPS, behavioral examinations and drug administration are shown in Figure 1.

**Figure 2.**
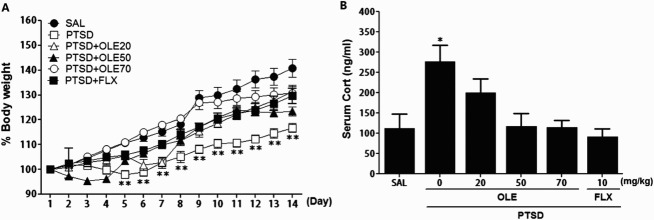
Effects of OLE administration on body weights gain (A) and serum CORT levels (B) in rats exposed to SPS. *p < 0.05, **p < 0.01 *vs*. SAL group.

### Elevated plus maze test

Animals exhibiting anxiety-like behavior typically experience a reduced number of entries and amount of time spent in the open arms of the maze and, thus, an increased amount of time in the closed arms of the maze. This apparatus used in the present study consisted of two open arms (50 × 10 cm each), two closed arms (50 × 10 × 40 cm each) and a central platform (10 × 10 cm) arranged such that the open arms and closed arms were directly opposite to each other. The EPM apparatus was constructed from black Plexiglas and elevated 50 cm above the floor. Exploration of the open arms was encouraged by testing under indirect dim light (2 × 60 W). At the beginning of each trial, the animals were placed in the center of the maze facing towards the closed arm, and the following parameters were recorded during the 5-min test period: number of open-arm entries, number of closed-arm entries, time spent in open arms, and time spent in closed arms. Anxiety reduction was defined as an increase in the number of entries into the open arms relative to total entries into both the open and closed arms, and total arm entries were used as an indicator of changes in locomotor activity. Anxiety index was calculated as follows:

$$\begin{array}{rl} & \text{Anxiety index}\\ & =1-\left[\left(\frac{\text{Time spent in the open arms}}{\text{Total time on the maze}}\right)\frac{+\left(\frac{\text{Number of entries to the open arms}}{\text{Total exploration on the maze}}\right)}{2}\right] \end{array}$$

Anxiety index values range from 0 to 1 where an increase in the index expresses increased anxiety-like behavior (Serova et al. 2013). Unprotected head dips were defined as peering over the edge of an open arm with head, neck, and shoulders. Grooming behavior has been reported to be a response to novelty and other stressors. Because appropriate examination grooming behavior in EPM requires longer than 5 min session, duration (total time spent for grooming) and frequency (number of grooming bouts) were scored during 10 min in the EPM (Serova et al. 2013).

### Open field test

After the completion of EPM test, the animals were subjected to the open field test. The open filed area consisted of an enclosed square arena made of dark opaque Plexiglas (60 × 60 cm) surrounded by walls (30 cm high). The arena was divided by transverse lines into 20 × 20 equal squares (total 9 squares). All locomotor activities (animal's movements) was measured by a video camera mounted above the center of the maze. The open field maze was divided into two zones, central and peripheral zone, using the square drawn on the maze. The rats were placed in one corner of the open field and its activity during the subsequent 5 min was assessed. Locomotion (central zone crossing) and time spent in central and peripheral zone were observed.

### Corticosterone, serotonin (5-HT) and norepinephrine (NE) measurement

After PTSD for 14 days, CORT concentration in blood, and 5-HT and NE concentration in brain tissue were determined. The CORT concentration was measured by a competitive enzyme-linked immunoassay (ELISA) using a rabbit polyclonal CORT antibody (Corticosterone ELISA kit; Novus Biologicals, LLC., Littleton, CO, USA) according to the manufacturer's protocol. The 5-HT and NE concentration was measured by a competitive enzyme-linked immunoassay (ELISA) using a mouse monoclonal 5-HT antibody (5-HT ELISA kit; Abcam, Cambridge, MA, USA) and a mouse monoclonal NE antibody (NE ELISA kit; Novus Biologicals, LLC., Littleton, CO, USA) according to the manufacturer's protocol. All reaction products were measured at 450 nm using an ELISA reader (MutiRead 400; Authos Co., Vienna, Austria), and their amounts were calculated in ng/mL from standards.

### Immunohistochemical analyses of neuropeptide Y (NPY) and tyrosine hydroxylase (TH)

To conduct the immunohistochemical analyses, three rats from each group were deeply anesthetized with sodium pentobarbital (80 mg/kg, i.p.) and perfused through the ascending aorta with 0.9% saline followed by 300 ml 4% paraformaldehyde in 0.1 M phosphate-buffered saline (PBS). The brains were removed in a randomized order, post-fixed overnight, and cryoprotected with 20% sucrose in 0.1 M PBS at 4°C. Coronal sections (30 μm) were cut through the hippocampus and locus coeruleus (LC) using a cryostat. Briefly, the sections were incubated with primary rabbit anti-NPY antibody (1:2000 dilution, Immunostar, Hudson, WI, USA) and primary sheep anti-TH antibody (1:2000 dilution, Chemicon International Inc., Temecula, CA, USA) for 72 h at 4°C. Next, the sections were incubated for 120 min at room temperature with secondary antibodies (1:200 dilution, Vector Laboratories Co., Burlingame, CA, USA) in PBST containing 2% normal serum. To visualize immunoreactivity, the sections were incubated for 90 min in ABC reagent (Vectastain Elite ABC kit, Vector Labs. Co.), and then in a solution containing 3,3′-diaminobenzidine (DAB; Sigma) and 0.01% H_2_O_2_ for 1 min. The sections were viewed at 200× magnification, and the numbers of NPY and TH labeled cells was quantified in the hippocampus and LC.

### Total RNA preparation and reverse transcription-polymerase chain reaction

The mRNA expression levels of brain-derived neurotrophic factor (BDNF) in hippocampal tissues isolated from four rats per group were determined using reverse transcription**-**polymerase chain reaction (RT**-**PCR). Following sacrifice, the brains were removed quickly and stored at −80°C until use. Total RNA was prepared from the brain tissue samples using TRIzol® reagent (Invitrogen Co., Carlsbad, CA, USA) according to the supplier's instructions. Briefly, complementary DNA was synthesized from total RNA using reverse transcriptase (Takara Co., Shiga, Japan). The PCR products were separated on 1.2% agarose gels and stained with ethidium bromide, and the density of each band was quantified using an image-analyzing system (i-Max™, CoreBio System Co., Seoul, Korea). The expression levels of each were compared by calculating the relative density of the target band, such as BDNF, with that of GAPDH.

### Statistical analysis

All measurements were performed by an independent investigator blinded to the experimental conditions, and the results are expressed as means ± standard error of the mean (SE). Differences within or between normally distributed data were analyzed using an analysis of variance (ANOVA) using SPSS (Version 13.0, SPSS, Inc., Chicago, IL, USA) and a Tukey's post hoc test. In all of the analyses, the differences were considered statistically significant at *p* < 0.05.

## Results

### Effects of OLE on changes in body weight and serum CORT levels following SPS exposure

Compared with the saline-treated (SAL) group, the rats in the SPS group showed a significant gradual reduction in body weight gain during 10 days (*p* < 0.01) (Figure 2A). However, rats treated with 20, 50, or 70 mg/kg of OLE, didn't showed significant difference in reduction of body weight gain compared with the SPS group.

An ELISA revealed that SPS increased serum CORT concentrations significantly, by 240.51%, compared with saline treatment (*p* < 0.05) (Figure 2B). However, the administration of OLE inhibited the SPS-induced increase in serum CORT levels, although this result was only minimally statistically significance.

### Effects of OLE on anxiety-like behavior following SPS

**Figure 3.**
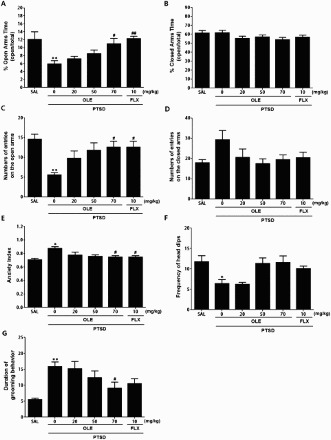
Effects of OLE administration on the percentage of time spent in the open (A) and closed (B) arms, numbers of entries into open (C) and closed (D) arms, anxiety index (E), unprotected head dips (F) and grooming behavior (G) in the EPM test of rats exposed to SPS. *p < 0.05, **p < 0.01 *vs*. SAL group; ^#^*p* < 0.05, ^##^*p* < 0.01 *vs*. PTSD group.

Post hoc comparisons revealed a significant decrease in the percentage of time spent in the open arms of the maze by rats exposed to SPS for 14 days versus saline-treated rats were not exposed to stress (*p* < 0.01; Figure 3A). However, the rats in the PTSD+OLE70 group were showed significant restoration of the percentage of time spent in the open arms of the maze, which had previously decreased following SPS exposure, compared with the PTSD group (*p* < 0.05). There was no significant difference in the percentage of time spent in the closed arms among the groups (Figure 3B). Similarly, post hoc comparisons revealed a significant decrease in the number of entries in the open arms of the maze in rats exposed to SPS for 14 days compared with the SAL group (*p* < 0.01; Figure 3C). Rats in the PTSD+OLE70 group entered the open arms of the EPM significantly more often than rats in the PTSD group (*p* < 0.05). Because there was no significant difference in the number of entries in the closed arms among the groups, the observed anxiety-like behaviors of the rats exposed to SPS were likely not attributable to differences in their locomotor activity (Figure 3D). Overall, the anxiety index, calculated based on the number of visits to and time spent in the open and closed arms was also different in the six groups of rats with lower values in the OLE-treated rats (*p* < 0.05; Figure 3E). OLE administration significantly decreased the frequency of unprotected head dips compared with the PTSD group, although this result was only minimally statistically significant (Figure 3F). The total time, and duration of a single bout of grooming behavior in the EPM test are shown in Figure 3G. The duration of grooming behavior was modified only by 70 mg of OLE given prior to SPS exposure (*p* < 0.05).

**Figure 4.**
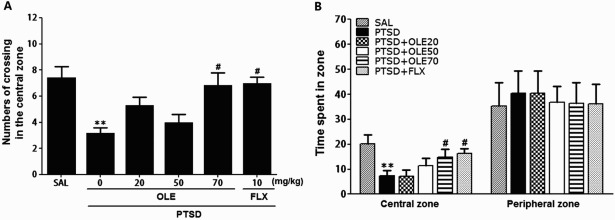
Effects of OLE administration on locomotion and exploratory behavior in the OFT of rats exposed to SPS. Change in the numbers of crossing in the central zone (A), and the time spent in central zone and peripheral zones (B). **p < 0.01 *vs*. SAL group; ^#^*p* < 0.05 *vs*. PTSD group.

### Effects of OLE on spontaneous locomotion and exploratory behavior following SPS

Rats exposed to SPS significantly decreased the time spent in the central zone with a corresponding increase in time spent in the peripheral zone versus the SAL group (*p* < 0.01) (Figure 4B). There was also a significant decrease in the number of central zone crossings following the SPS procedure (*p* < 0.01) (Figure 4A). However, the OLE-treated rats (70 mg/kg) displayed a significant increase the time spent and number of central zone crossings compared with the PTSD group (*p* < 0.05).

### Effects of OLE on the 5-HT and NE concentrations in the hippocampus following SPS

**Figure 5.**
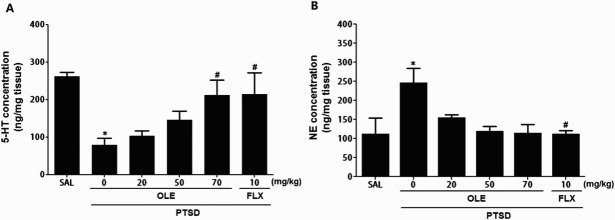
Effects of OLE administration on the 5-HT (A) and NE (B) concentrations in the hippocampus of rats exposed to SPS. *p < 0.05 *vs*. SAL group; ^#^*p* < 0.05 *vs*. PTSD group.

ELISAs analysis demonstrated that rats exposed to SPS for 14 days showed significantly decreased 5-HT and increased NE concentrations in the hippocampus by 29.96% and 253.14%, respectively versus rats in the untreated PTSD group (*p* < 0.05; Figure 5). Daily administration of OLE decreased the SPS-induced increase in NE concentration in the hippocampus as compared with the SPS group, although this result was only minimally statistically significant. Also, OLE administration of OLE significantly increased the SPS-induced decrease in 5-HT concentration in the hippocampus as compared with the PTSD group (*p* < 0.05).

### Effects of OLE on NPY- and TH-like immunoreactivities following SPS

**Figure 6.**
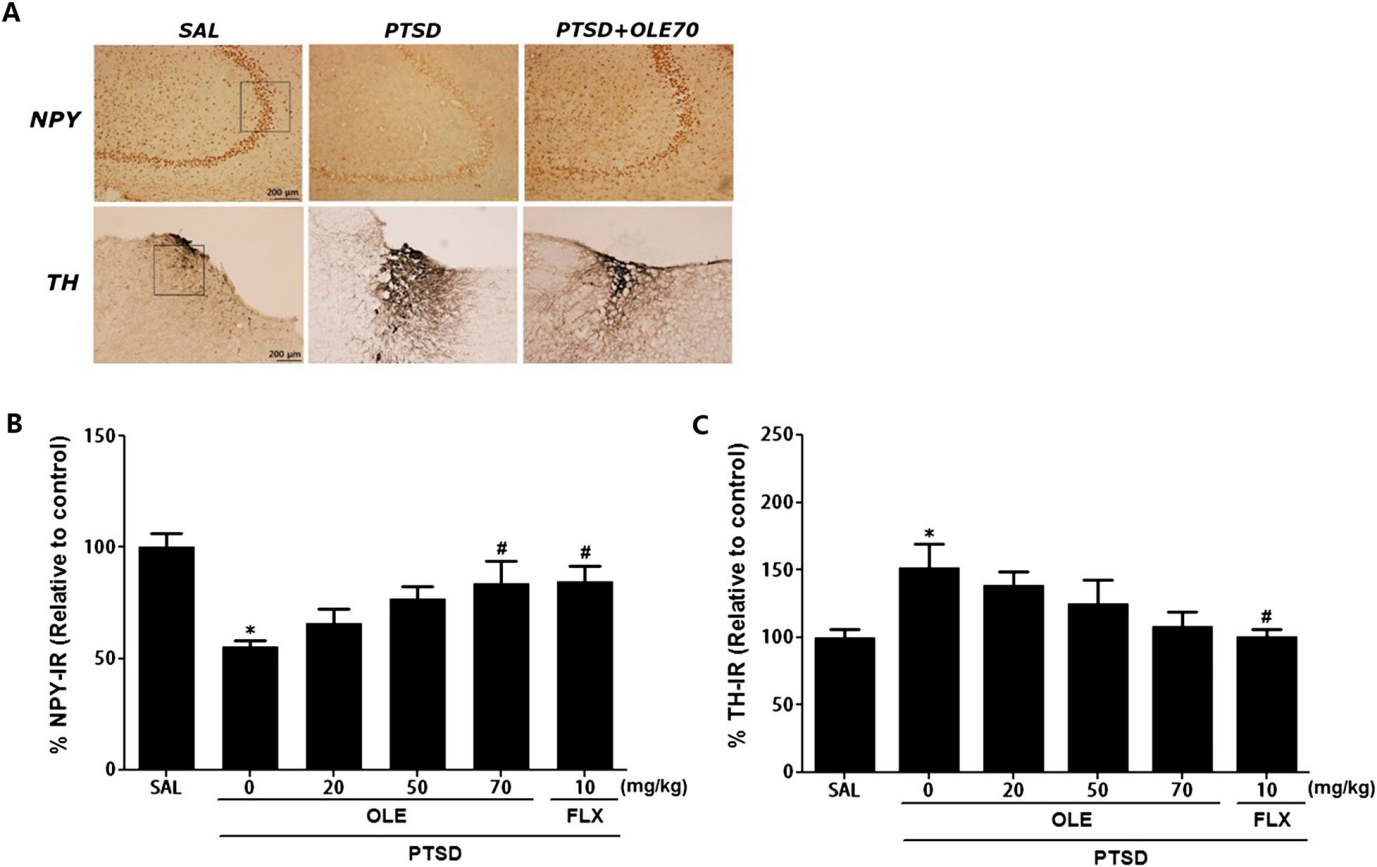
Effects of OLE administration on the level of NPY expression in the hippocampus and TH expression in the LC of rats exposed to SPS. Representative images (A) and the relative percentage values (B and C) are shown. *p < 0.05 *vs*. SAL group; ^#^*p* < 0.05 *vs*. PTSD group.

Compared with the SAL group, NPY-like immunoreactive cells in the hippocampus of the PTSD group decreased, to 55.28%, indicating that SPS resulted in a significant decrease in NPY expression. Compared with the PTSD group, there was a significant increase in the number of NPY-immunoreactive neurons in the hippocampus region of the rats in the PTSD+OLE70 group (*p* < 0.05; Figure 6A and B). Compared with the SAL group, TH-immunoreactive cells in the LC of the PTSD group increased, by 151.47%, indicating that SPS resulted in a significant increase in TH expression (*p* < 0.05; Figure 6A and C). Compared with the PTSD group, there was a slight decrease in the number of TH-immunoreactive neurons in the LC of the PTSD+OLE70 group; however, this result was only minimally statistically significant.

**Figure 7.**
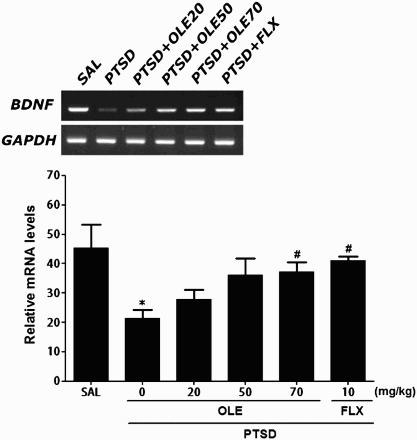
Effects of OLE administration on the expression of BDNF mRNA in rats exposed to SPS for 14 consecutive days. PCR product bands on an agarose gel and their relative intensities are shown. *p < 0.05 *vs*. SAL group; ^#^*p* < 0.05 *vs*. PTSD group.

### Effects of OLE on BDNF mRNA in the hippocampus following SPS

BDNF mRNA expression levels were normalized against those of GAPDH mRNA (internal control). There was a significant decrease in hippocampal BDNF mRNA expression in the PTSD group compared with the SAL group (*p* < 0.05) (Figure 7). The decreased expression of BDNF mRNA in the PTSD group was significantly restored to levels similar to those in the SAL group by 70 mg/kg of OLE (*p* < 0.05).

## Discussion

The administration of OLE after SPS exposure significantly increased the number and duration of open-arm visits in the EPM test, reduced the anxiety index, and decreased grooming behavior. The administration of OLE after SPS also significantly reduced anxiety-like behavior, as indicated by an increase in central zone crossings in the OFT. Thus, OLE appears to act as an anxiolytic. These results could lead to the development of novel therapeutics for the treatment of PTSD.

The results of our behavioral investigations demonstrate the anxiolytic-like effects of OLE in an animal model of anxiety. The administration of OLE after SPS significantly reduced anxiety-like behavior in the EPM test, as indicated by more entries and exploratory behaviors, and less avoidance of the open arms, yielding lower anxiety indices (Cohen et al. 2012). OLE administration after SPS also significantly increased the time spent and number of central zone crossings in the OFT. These changes in anxiety-like behavior appear to have been specific and not the result of impaired locomotor activity because the track length in the EPM test and OFT was similar for all groups. Thus, because behavior in the EPM test and OFT are related to HPA axis-associated psychological dysfunction, these results indicate that inhibition by OLE may attenuate HPA axis activity.

In the present study, in rats subjected to SPS, OLE reduced the decrease in NPY expression in the hippocampus of rats subjected to SPS (Serova et al. 2014). SPS-induced psychopathological conditions, including anxiety, have been linked to TH activity, so we assessed TH expression in the LC and found decreased TH-like immunoreactivity (Serova et al. 2013). Some studies suggest that SPS results in physiological and behavioral changes reminiscent of PTSD symptoms linked to the LC-NE system, and SPS is known to induce an elevation in TH mRNA in the LC (George et al. 2013). However, OLE did not significantly inhibit the increase in expression of TH in the LC or hippocampal NE levels. Many studies have suggested that PTSD patients have elevated NE levels in the brain and that this increase is correlated with disease severity (Geracioti et al. 2008). Exaggerated central noradrenergic activity is primarily observed in response to various trauma-associated stimuli rather than under resting conditions, in PTSD patients (Geracioti et al. 2008). Thus, OLE may not modulate the central adrenergic system, and may indirectly alter catecholamine synthesis in the brain. Many studies have suggested that PTSD can affect the neuronal circuitry and cause an imbalance in neurotransmitters, intensifying anxiety (Ebenezer et al. 2016). We hypothesized that SPS-induced anxiety-like symptoms would be associated with central 5-HT dysfunction. We have shown that PTSD animals treated with OLE had significant increases in 5-HT levels in the hippocampus and that this may have inhibited the pathophysiology of PTSD. The effects of OLE can be reversed by 5-HT manipulation (Lin et al. 2016). Thus, the present findings indicate that OLE, like FLX, attenuates the behavior and neurochemical responses associated with anxiety by modulating NPY expression and the serotonergic system in the brain (Aykaç et al. 2012).

Our study also found that SPS decreased the expression of BDNF mRNA in rat hippocampus as well as anxiety-like behaviors. OLE restored hippocampal BDNF mRNA levels, suggesting that the modulation of BDNF signaling plays a role in the anxiolytic actions of OLE.

In summary, OLE administration was associated with anxiolytic-like effects in the EPM test and OFT, possibly via modification of NPY expression in the serotonergic system. These findings indicate that OLE can ameliorate the complex behavior and neurochemical responses involved in anxiety. Thus, OLE may be a useful agent in preventing anxiety-like behavior associated with PTSD.
